# A Simplified 4-Site Economical Intradermal Post-Exposure Rabies Vaccine Regimen: A Randomised Controlled Comparison with Standard Methods

**DOI:** 10.1371/journal.pntd.0000224

**Published:** 2008-04-23

**Authors:** Mary J. Warrell, Anna Riddell, Ly-Mee Yu, Judith Phipps, Linda Diggle, Hervé Bourhy, Jonathan J. Deeks, Anthony R. Fooks, Laurent Audry, Sharon M. Brookes, François-Xavier Meslin, Richard Moxon, Andrew J. Pollard, David A. Warrell

**Affiliations:** 1 The Oxford Vaccine Group, Centre for Clinical Vaccinology & Tropical Medicine University of Oxford, Churchill Hospital, Oxford, United Kingdom; 2 Centre for Statistics in Medicine, University of Oxford, Oxford, United Kingdom; 3 UPRE Lyssavirus Dynamics and Host Adaptation, WHO Collaborating Centre for Reference and Research on Rabies, Institut Pasteur, Paris, France; 4 Rabies and Wildlife Zoonoses Group, WHO Collaborating Centre for the Characterization of Rabies and Rabies-Related Viruses, Veterinary Laboratories Agency, Weybridge, United Kingdom; 5 Zoonoses and VPH, Department of Food Safety, Zoonoses and Foodborne Diseases, World Health Organization, Geneva, Switzerland; The George Washington University, United States of America

## Abstract

**Background:**

The need for economical rabies post-exposure prophylaxis (PEP) is increasing in developing countries. Implementation of the two currently approved economical intradermal (ID) vaccine regimens is restricted due to confusion over different vaccines, regimens and dosages, lack of confidence in intradermal technique, and pharmaceutical regulations. We therefore compared a simplified 4-site economical PEP regimen with standard methods.

**Methods:**

Two hundred and fifty-four volunteers were randomly allocated to a single blind controlled trial. Each received purified vero cell rabies vaccine by one of four PEP regimens: the currently accepted 2-site ID; the 8-site regimen using 0.05 ml per ID site; a new 4-site ID regimen (on day 0, approximately 0.1 ml at 4 ID sites, using the whole 0.5 ml ampoule of vaccine; on day 7, 0.1 ml ID at 2 sites and at one site on days 28 and 90); or the standard 5-dose intramuscular regimen. All ID regimens required the same total amount of vaccine, 60% less than the intramuscular method. Neutralising antibody responses were measured five times over a year in 229 people, for whom complete data were available.

**Findings:**

All ID regimens showed similar immunogenicity. The intramuscular regimen gave the lowest geometric mean antibody titres. Using the rapid fluorescent focus inhibition test, some sera had unexpectedly high antibody levels that were not attributable to previous vaccination. The results were confirmed using the fluorescent antibody virus neutralisation method.

**Conclusions:**

This 4-site PEP regimen proved as immunogenic as current regimens, and has the advantages of requiring fewer clinic visits, being more practicable, and having a wider margin of safety, especially in inexperienced hands, than the 2-site regimen. It is more convenient than the 8-site method, and can be used economically with vaccines formulated in 1.0 or 0.5 ml ampoules. The 4-site regimen now meets all requirements of immunogenicity for PEP and can be introduced without further studies.

**Trial Registration:**

Controlled-Trials.com ISRCTN 30087513

## Author Summary

All human deaths from rabies result from failure to give adequate prophylaxis. After a rabid animal bite, immediate wound cleaning, rabies vaccine and immunoglobulin injections effectively prevent fatal infection. Immunoglobulin is very rarely available in developing countries, where prevention relies on efficacious vaccine. WHO approved vaccines are prohibitively expensive, but 2 economical regimens (injecting small amounts of vaccine intradermally, into the skin, at 2 or 8 sites on the first day of the course) have been used for many years in a few places. Practical or perceived difficulties have restricted widespread uptake of economical methods. These could largely be overcome by introducing a new, simpler regimen, involving 4 site injections on the first day. We vaccinated volunteers to compare the antibody levels induced by the 4-site intradermal regimen with those induced by the current 2-site and 8-site regimens and the “gold standard” intramuscular regimen favoured internationally. All the economical intradermal regimens were at least as immunogenic as the intramuscular method. The results provide sufficient evidence that the 4-site regimen meets the criteria necessary for its recommendation for use wherever the cost of vaccine is prohibitive and especially where 2 or more patients are treated on the same day.

## Introduction

Rabies is a neglected disease affecting particularly tropical developing countries [1]. Estimates of the Global use of rabies post-exposure prophylaxis (PEP) are rising. In China, it was 8 million in 2005 [2], yet rabies currently kills more people than any other infectious disease there. Rabies deaths are underreported and misdiagnosed, for example as cerebral malaria [3]. As the obsolete nervous tissue-based rabies vaccines are replaced by expensive tissue culture vaccines, there is increasing need to reduce the cost of post-exposure prophylaxis. In Africa, the average cost of a standard intramuscular (IM) course of vaccine is $39.6, equivalent to 50 days wages [1]. There is a shortage of affordable rabies vaccines of reliable quality in the developing world [4].

Economical PEP regimens employ multiple site intradermal (ID) injections, saving 60% of the vaccine used in the standard IM method (Table 1). Increasing the number of sites of injection is designed to stimulate several different groups of lymph nodes to initiate antibody production. Two economical regimens are now recommended [5], an 8-site [6] and a 2-site [7] method (Table 1). The urgency of PEP demands a rapid induction of neutralising antibody using minimal amounts of vaccine in all recipients including the ∼3% of the population who are ‘low responders’ [8] and the many others whose immune response is impaired [9]–[12].

**Table 1:** Vaccine Regimens Showing Number of Sites of Injection.

Regimen	Day 0	Day 3	Day 7	Day 14	Day 28	Day 90	Total Ampoules of Vaccine
A 4-site ID	4 (0.5 ml)*		2		1	1	1.8 (0.9 ml)
B 8-site† ID	8 (0.5 ml)*		4		1	1	1.6 (0.8 ml)
D 2-site ID	2 (0.2 ml)	2	2		1	1	1.6 (0.8 ml)
E IM	1 (0.5 ml)	1	1	1	1		5

*: Whole ampoule of vaccine divided between sites.

**†:** For the 8-site regimen, the intradermal (ID) dose is 0.05 ml per site. All other ID doses are 0.1 ml per site.

ID injection sites over deltoids for 1 or 2 sites. 4 sites are deltoids and thighs.

8 sites are deltoids, thighs, suprascapular, and lower anterior abdominal wall areas. Intramuscular (IM) injection into the left deltoid.

The original IM PEP vaccine regimen is the most widely used globally. In Asia only 3% of tissue culture rabies vaccine treatments use economical ID regimens [1], and they are rarely used in Africa . The reasons are misgivings about reducing the vaccine dosage in prevention of a fatal disease [13], confusion over regimens, and the competence of staff giving ID inoculation. Economical regimens require sharing of ampoules between patients, but rabies vaccines have no added preservative and so the reconstituted ampoule of vaccine should be used within a day. The use of economical regimens is therefore mainly confined to large treatment centres, yet 90% of rabies deaths occur in rural areas [4].

Evidence to date indicates that the 8-site regimen is more immunogenic than the 2-site regimen [14],[15]. However the 8-site method is not economical when used with one of the two major vaccines, purified vero cell rabies vaccine (PVRV) (Verorab™ Sanofi Pasteur), because this vaccine is relatively concentrated: an IM dose is 0.5 ml, in contrast to the equivalent 1 ml dose of the other widely used vaccine, purified chick embryo cell vaccine (PCECV) (Rabipur™; Novartis) [15]. Although the 8-site regimen has some advantages and was recommended by some authorities for use when rabies immunoglobulin (RIG) was not available [15], the 2-site regimen is more acceptable and convenient. The total dose of vaccine should be the same with the two regimens. The only difference between the two schedules is that with the 8-site a large dose of vaccine is given on the first day, whereas with the 2-site regimen this is divided between days 0 and 3, entailing an extra treatment visit [14]. Ambrozaitis et al. [16] demonstrated that the 4-site regimen was apparently immunogenic with both PVRV and PCEC vaccines, but there was no comparison with any current PEP method and historical controls are unreliable.

For all these reasons, a single, simple, acceptable, immunogenic and economical PEP regimen is needed, suitable for use with all vaccines fulfilling WHO requirements. We tested a 4-site PEP regimen which allows the 8-site regimen principle to be used economically with PVRV. We also investigated whether injecting the same amount of vaccine between 4 instead of 8 sites affected immunogenicity. The new 4-site regimen and the currently used ID regimens were compared with the standard IM method in a single blind, randomised, controlled trial.

## Methods

The CONSORT checklist for this study is available in Supporting Information as Checklist S1.

### Participants

Healthy volunteers were recruited in Oxford and Bristol UK, between June 2002 and April 2005. The exclusion criteria were: previous rabies vaccine treatment; pregnancy; a recent blood transfusion; taking immunosuppressive drugs; receiving another killed vaccine or chloroquine treatment [17] within 2 weeks, or any live virus vaccine within 3 weeks of a rabies vaccine dose. The Oxfordshire Clinical Research Ethics Committee approved the project (ref. C01.078), conducted in accordance with GCP regulations (EU Directive 2001/20/EC).

### Randomisation

Each participant was allocated to one of four rabies PEP regimens according to a computer generated list with fixed blocks of 12. Group A received the 4-site regimen; group B, the 8-site regimen; group D, the 2-site regimen and group E, the IM regimen. Allocations were concealed in opaque serially numbered sealed envelopes, opened once written informed consent had been obtained. All laboratory staff were blinded to the treatment allocation.

### Vaccine and regimens

The vaccine used was PVRV, (Verorab™ Sanofi Pasteur) Lot no XO291-1 potency 5.3 IU/dose in 165 subjects, and Lot no. U0271 potency 8.4 IU/dose in 64 subjects. The Medicines and Healthcare products Regulatory Agency granted exemption from a licence.

The 2-site and IM regimens were according to standard methods (Table 1) [15]. For the 4-site regimen, on day 0 the entire contents of the 0.5 ml PVRV vial are injected ID, divided between 4 sites over the deltoids and thighs (approximately 0.1 ml per site).

On day 7, 0.1 ml is injected ID at 2 sites (deltoids). Single site injections are given on days 28 and 90. The 8-site regimen is the exact equivalent of the current 8-site method [6],[15], using a vaccine containing 0.5 ml/ampoule. The entire contents of the vial are divided between 8 ID sites on day 0: (deltoids, thighs, suprascapular, lower anterior abdominal wall). The dose per site is approximately 0.05 ml. All the ID regimens use the same total amount of vaccine. There is a little inevitable wastage in syringes. Opened ampoules were refrigerated and used or discarded within 8 hours. See Table 1 for the timing, doses, routes and sites of inoculation of all the regimens.

### Serology

Blood samples were taken at days 0, 7, 14, 90 and 1 year. Serum aliquots were coded, stored at −70°C and assayed blind. Neutralising antibody levels were measured by an adaptation of the rapid fluorescent focus inhibition test (RFFIT) for 96 well plates [18],[19], at the Institut Pasteur, Paris. Briefly, a constant dose of challenge virus standard (CVS) is incubated with diluted test sera. An in-house reference serum (SHR2 31/03/06 = 22 IU/ml), is calibrated against an international standard (RAI = 30 IU/ml). Serum/virus mixtures are incubated, and BSR cells (a clone of BHK-21 cells) were added. After 24 hours incubation, the monolayer is acetone-fixed and stained with a fluorescent anti-nucleocapsid antibody (Chemicon). The result in IU/ml was the mean of independent duplicate tests.

Selected sera were also assessed using the fluorescent antibody virus neutralisation (FAVN) assay at the Veterinary Laboratories Agency ,Weybridge [20],[21]. This test is the same in principle as the RFFIT, using the same challenge virus. The FAVN and RFFIT vary in that they use a different dilution series (3 fold versus 5 fold); the FAVN runs samples in quadruplicate; BHK-21 cells (ATCC, USA) are used, and the internal serum standard is the WHO human positive control (NIBSC, UK). The antibody titre is based on 100% virus neutralisation for the FAVN and 50% reduction of fluorescent foci in the RFFIT.

Protocol deviations were not permitted on days 7 and 14, but flexibility was allowed if necessary: on day 28±1 day; on day 90 - 7 to +10 days, and at one year −2 weeks to +4 weeks. All records were kept in strict confidence. Volunteers kept a health record diary for a week after each vaccine dose.

### Statistical methods

The aim was to demonstrate that the 4-site test regimen was at least as immunogenic as the standard regimens. The primary outcome is the proportion of participants reaching the WHO criterion for post-exposure regimens: a minimum neutralising antibody level of 0.5 IU/ml by day 14. The failure rate for the current regimen in meeting this threshold is less than one in 1000. At this rate, the expected number of failures in the control group is likely to be zero. The sample size calculation was based on the assumption that the new regimen was just as effective (i.e. rate of less than 1 in 1000) and was computed by simulation method using exact methods for estimating the confidence interval (CI) for the difference.

The initial protocol envisaged the recruitment of 75 participants per group to make 5 comparisons over 7 regimens expecting zero events to be observed, giving 90% power to show that the difference in failure rates was at most 6.2% (adjusting for pre-planned multiple comparisons). Because the trial failed to recruit at an adequate rate, the revised sample size of 55 participants per group (Protocol S1) was calculated for a total of 6 comparisons among four groups giving 90% power to show that the difference in failure rates was at most 9% by day 14.

Proportions and 95% CI for the difference in proportions were calculated using the method based on Wilson's score [22]. Agreement between the results of the two antibody tests was assessed by the Bland-Altman method [23]. Titre concentrations were log transformed and groups were compared using analysis of variance. Results were deemed statistically significant at P<0.05. Fisher's exact test was used to compare side effects between groups. Post-hoc pairwise comparisons were also carried out on any local reactions (redness, swelling hardness, or tenderness/pain) and on any local or generalised signs or symptoms. P-values were adjusted if multiple comparisons were performed.

## Results

### Participant flow

**Figure 1:**
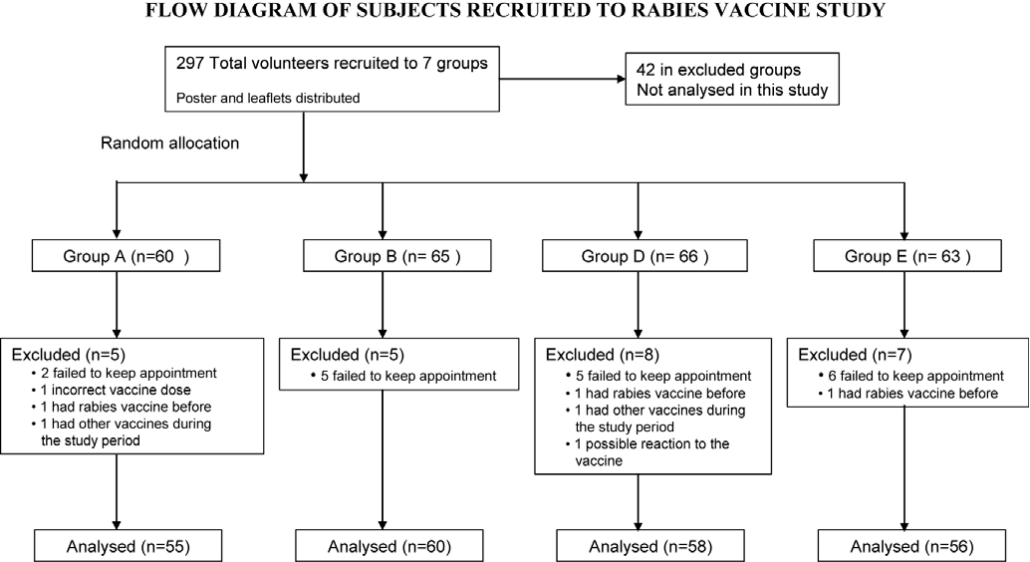
Flow diagram of subjects recruited to rabies vaccine study. Group A received the 4-site regimen; group B, the 8-site regimen; group D, the 2-site regimen and group E, the IM regimen.

Two hundred and fifty four subjects were recruited. Data from 229 were complete up to day 90, and used in the final analysis. Twenty five were excluded, usually because they failed to keep appointments (for details see Figure 1). Three sera taken on day 14 were lost during storage.

Subjects were aged between 18 and 50 years. Ages and sex ratios were similar between the groups (Table 2).

**Table 2:** Demographic Characteristics.

	Group A 4-site (n = 55)	Group B 8-site (n = 60)	Group D 2-site (n = 58)	Group E IM (n = 56)
Sex				
No. Females (%)	34 (61.8)	40 (66.7)	46 (79.3)	32 (57.1)
No. Males (%)	21 (38.2)	20 (33.3)	12 (20.7).	24 (42.9)
Ages (years)				
Mean (SD)	28.0 (9.2)	28.8 (9.6)	26.5 (6.9)	31.6 (10.0)
Range	18.3–50.8	19.0–49.4	18.1–44.4	18.1–50.7

### Side effects

One person withdrew within the first week because of transient arthralgia, possibly related to the vaccine.

Local reactions to the vaccine observed by 229 volunteers are shown in Table 3. Redness (erythema), swelling (inflammation) and hardness (induration) were more frequent in all ID groups than in the IM group (E) (P<0.0001). The incidence of local tenderness or pain was similar in all groups. Itchiness and local lymphadenopathy (tenderness at regional lymph nodes) was not solicited, but was volunteered more often in the ID groups (P<0.0001).

**Table 3:** Local Reactions.

Reaction	Group A 4-site (n = 55)	Group B 8-site (n = 60)	Group D 2-site (n = 58)	Group E IM (n = 56)	P-value
Redness	53 (96.4%)	58 (96.7%)	54 (93.1%)	14 (25.0%)	<0.0001
Swelling	42 (76.4%)	48 (80.0%)	46 (79.3%)	8 (14.3%)	<0.0001
Hardness	41 (74.6%)	44 (73.3%)	38 (65.5%)	9 (16.1%)	<0.0001
Tenderness/ Pain	39 (70.9%)	35 (58.3%)	41 (70.7%)	31 (55.4%)	0.2
Any above reactions	53 (96.4%)	58 (96.7%)	54 (93.1%)	33 (58.9%)	<0.0001*
Itchiness	26 (47.3%)	29 (48.3%)	28 (48.3%)	2 (3.6%)	<0.0001
Lymphadenopathy	5 (9.1%)	15 (25.0%)	8 (13.8%)	0 (0%)	<0.0001

*: Post hoc test: A vs E, P<0.0001; B vs E, P<0.001; D vs E, P<0.001.

Volunteers were asked to report any generalised symptoms, whether or not listed in their reaction diary. Some were attributable to causes unrelated to vaccination. The incidence of each of the generalised symptoms was lower with the IM regimen but this only reached significance when compared with all three ID groups together (P<0.001) (Table 4).

**Table 4:** Generalised Symptoms Reported and the % Possibly or Probably Related to Vaccine.

Reaction	Group A 4-site	Group B 8-site	Group D 2-site	Group E IM	P-value
Shivery	7/9 (78%)	5/11 (45%)	4/6 (67%)	3/6 (50%)	0.5
Vomited	2/2 (100%)	0/1 (0%)	0/3 (0%)	0/2 (0%)	0.07
Musc/joint	11/15 (73%)	13/25 (52%)	7/11 (64%)	4/12 (31%)	0.2
Headache	25/32 (78%)	23/39 (59%)	21/32 (66%)	9/22 (41%)	0.05
Diarrhoea	5/6 (83%)	2/4 (50%)	1/3 (33%)	0/1 (0%)	0.3
Rash	4/4 (100%)	4/4 (100%)	2/3 (67%)	1/1 (100%)	0.3
Any generalised symptoms	33/34 (97%)	31/34 (91%)	26/28 (93%)	13/14 (93%)	0.8
Any local or generalised signs or symptoms	55 (100%)	58 (96.7%)	54 (93.1%)	35 (62.5%)	<0.0001*

*: Post hoc test: A vs E, P<0.0001; B vs E, P<0.001; D vs E, P<0.001

### RFFIT serology results

The lower limit of detection of antibody was 0.06 IU/ml, while the threshold for a positive result, was 0.3 IU/ml, as naïve sera can range between 0 and 0.3 IU/ml.

Two sera gave pre-vaccination results above this threshold (the means of two tests were 0.38 IU/ml and 0.46 IU/ml). These subjects denied previous rabies immunisation and subsequent titres did not suggest a secondary immune response, but they were excluded from the analysis. Data for the remaining 227 people were analysed. Undetectable titres were assigned the value of 0.02.

**Figure 2:**
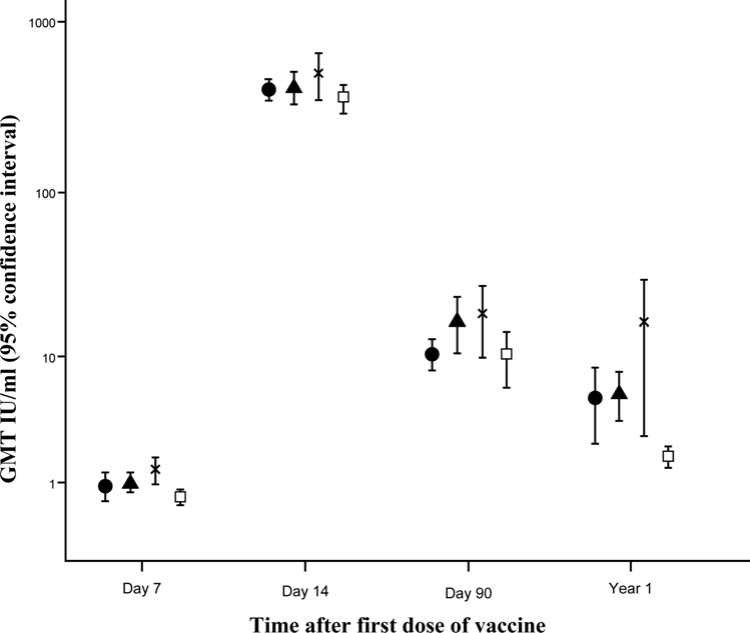
Rabies neutralising antibody results by the rabies fluorescent focus inhibition test (RFFIT). Symbols represent: • Group A 4-site; ▴ Group B 8-site; x Group D 2-site; □ Group E intramuscular (IM). Points are geometric mean titres with 95% confidence intervals.

Geometric mean titres (GMTs) on day 7 for the 4 treatment groups (Table 5, Figure 2), showed that group E (IM) had a lower GMT than group D (2-site) (P<0.001) and group B (8-site) (P = 0.01). Group A (4-site) was lower than group D (P = 0.01). The percentage of people with detectable antibody >0.3 IU/ml was 60%, 77.6%, 86.2% and 62.5% for groups A, B, D and E respectively. The day 7 results were no different with the two batches of vaccine (data not shown).

**Table 5:** RFFIT Serology Results.

	Group A 4-site	Group B 8-site	Group D 2-site	Group E IM
Day 7				
N	55	58	58	56
GMT*	0.44	0.67	0.88	0.36
Range	(0.02 to 6.89)	(0.06 to 6.21)	(0.2 to 8.39)	(0.02 to 2.79)
95% CI	(0.31 to 0.61)	(0.53 to 0.86)	(0.69 to 1.11)	(0.26 to 0.50)
Day 14				
N	54	58	58	54
GMT*	334.66	308.11	363.66	228.45
Range	(43.7 to 811.5)	(24.3 to 1459.0)	(60.9 to 3711.5)	(5.5 to 1282.6)
95% CI	(278.68 to 401.79)	(248.66 to 381.77)	(299.16 to 442.08)	(161.99 to 322.18)
Day 90				
N	55	56	58	56
GMT*	7.18	9.75	9.14	6.21
Range	(0.9 to 29.5)	(0.9 to 153.1)	(1.2 to 228.9)	(0.9 to 81.9)
95% CI	(5.63 to 9.15)	(7.46 to 12.76)	(6.86 to 12.20)	(4.86 to 7.95)
1 year				
N	44	50	55	53
GMT*	2.52	3.21	4.60	1.33
Range	(0.35 to 58.34)	(0.36 to 31.71)	(0.62 to 295.17)	(0.10 to 6.32)
95% CI	(1.79 to 3.55)	(2.38 to 4.34)	(3.31 to 6.42)	(1.05 to 1.69)

*: IU/ml.

**Figure 3:**
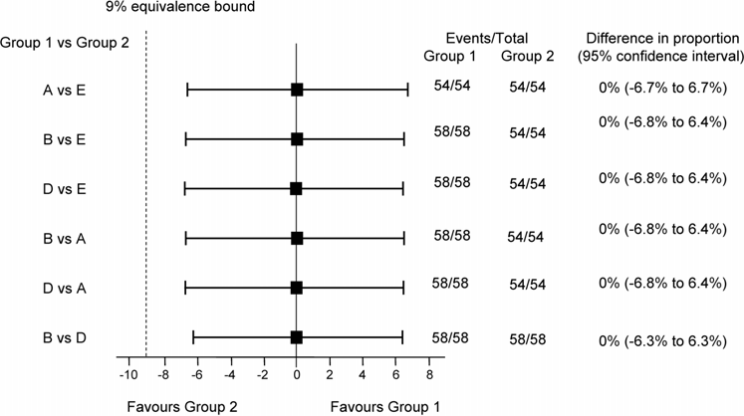
Day 14 rabies fluorescent focus inhibition test (RFFIT) results antibody levels ≥0.5 IU/ml: difference in proportion between the geometric mean titres (GMTs) of any two regimens. Group A = 4-site intradermal (ID); Group B = 8-site ID; Group D = 2-site ID; Group E = intramuscular.

On day 14 all subjects had antibody levels >0.5 IU/ml (Table 5, Figure 2). The 95% confidence intervals for the differences in proportions between any two regimens indicated that differences could at most be between 6% and 7% (Figure 3). The only significant difference between the GMTs is that Group E (IM) was lower than group D (2-site) (P = 0.04).

On day 90, GMTs were similar (Table 5, Figure 2). At 1 year all ID recipients had detectable antibody, but two people in group E (IM) had <0.3 IU/ml. Eight had levels between 0.3 and 0.5 IU/ml: 2 in group A, 2 in group B and 6 in group E. All the ID regimens induced more persistent antibody than the IM group (P<0.001 for groups B and D, P<0.02 for A). The 2-site (D) GMT was greater than the 4-site (A) (P<0.04).

Before the serological data were decoded, some unusual results were identified. Antibody levels were unexpectedly high on days 7 and 14, compared with other clinical trials [6],[7],[16]. Two subjects were excluded because they had pre-vaccination antibody levels above the 0.3 IU/ml threshold. Two people had antibody levels >3000 IU/ml on day 14 (both of them later proved to be in group D). The next highest were seven subjects with levels between 1000 and 1500 IU/ml. The other results for these people were well within the range of the rest. After decoding, none of the high results was found to be among group A subjects. They were individual high titres, without any suggestion of an anemnestic response. To confirm the results of the trial, 224 (of the original 229) day 7 samples available, and a few others (see below) were tested blind in another laboratory which uses the FAVN method.

### FAVN serology results and comparison with RFFIT

The FAVN lower limit of detection of antibody was 0.05 IU/ml. The threshold for a positive result is >0.13 as naïve sera can range up to 0.1 IU/ml.

**Figure 4:**
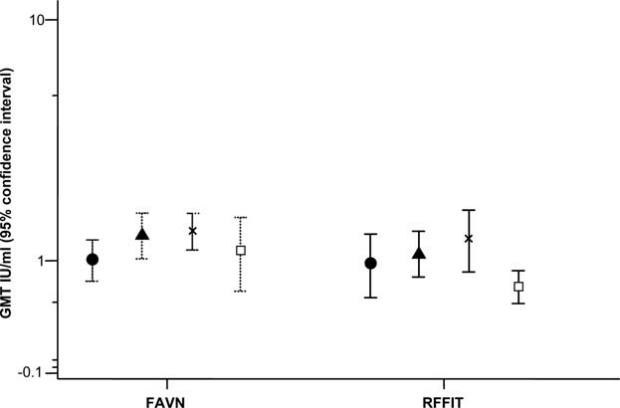
Rabies fluorescent focus inhibition test (RFFIT) results of the day 7 sera plotted with the results of the same sera tested by the fluorescent antibody virus neutralisation (FAVN) method. Symbols represent: • Group A 4-site; ▴ Group B 8-site; x Group D 2-site; □ Group E IM. Points are geometric mean titres with 95% confidence intervals.

The day 7 results for 224 subjects, including the 2 excluded because of high initial RFFIT titres, showed GMTs between 1.044 IU/ml for group D (2-site) and 0. 573 IU/ml for E (IM) (P<0.01) (Table 6, Figure 4). There were no other significant differences and GMTs were in the same order as the RFFIT results for all 4 treatment groups. The percentage of people with detectable antibody, >0.13 IU/ml, was 96.3 %, 93.0%, 96.6 % and 87.3 % for groups A, B, D and E respectively.

**Table 6:** FAVN Serology Results for Day 7.

	Group A 4-site	Group B 8-site	Group D 2-site	Group E IM
N	54	57	58	55
GMT*	0.673	0.907	1.044	0.573
Range	(0.13 to 4.50)	(0.10 to 5.92)	(0.10 to 5.92)	(0.06 to 13.5)
95% CI	(0.52 to 0.87)	(0.70 to 1.18)	(0.82 to 1.32)	(0.42 to 0.78)

*: IU/ml.

**Figure 5:**
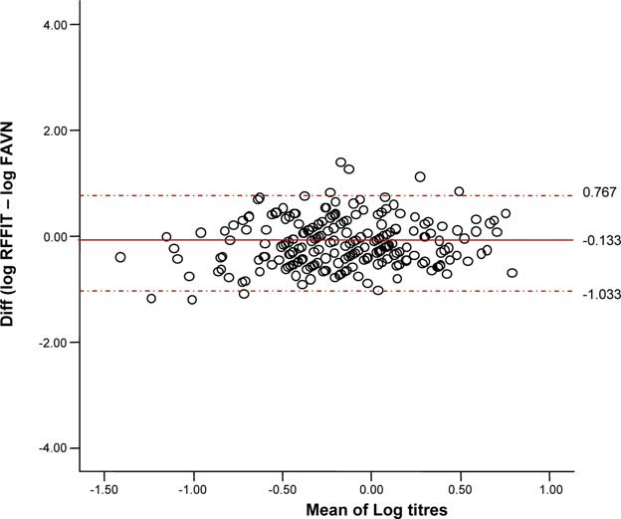
Bland-Altman plot of the difference between log RFFIT and log FAVN results, and the mean of the two values. Dotted lines are the 95% agreement limit. RFFIT = rabies fluorescent focus inhibition test, FAVN = fluorescent antibody virus neutralisation.

This comparison showed general consistency but considerable individual variation, as demonstrated graphically in a Bland-Altman plot (Figure 5). Further analysis was not appropriate in such a small sample. All the FAVN results were <6 IU/ml, except one of 13.5 IU/ml (the RFFIT result was 2.8 IU/ml). For the RFFIT, all titres were <7 IU/ml, except two of 8.39 and 9.36 IU/ml (the FAVN results were 1.14 and 3.42 IU/ml respectively).

The day 0 sera with RFFIT results >0.3 IU/ml, and day 14 sera with RFFIT results >3000 IU/ml, were included in a group of 38 otherwise randomly selected sera to be tested by the FAVN method. The day 0 results of 0.38 and 0.46 IU/ml were both 0.06 IU/ml by the FAVN. RFFIT results of 3711.5 and 3021.5 IU/ml were 53.3 and 121.2 IU/ml respectively by the FAVN.

## Discussion

### Interpretation

This study demonstrates that ID rabies vaccination is at least as immunogenic as the standard IM regimen and induces greater persistent immunity. ID regimens are therefore recommended anywhere in the world where the cost of PEP is critical. All three ID regimens required the same total amount of vaccine and proved equally immunogenic, but the 4-site ID regimen has several key advantages.

### Advantages over the 2-site regimen

First, the 4-site needs one less clinic visit (omitting day 3). WHO now recommends omitting the day 90 dose of ID regimens, and doubling the day 28 dose [24],[25],[26]. The 4-site regimen would then require only 3 visits (days 0, 7 and 28) the same as the current 3 dose IM **pre**-exposure regimen, but using only about half the amount of vaccine.

Secondly, the 4-site regimen is safer than the 2-site as it uses a whole ampoule of vaccine divided between intradermal sites on the crucial first day. If some vaccine were inadvertently injected subcutaneously, the wide margin of safety would ensure an adequate immune response [27]. Thirdly, sharing of ampoules of vaccine between patients is only necessary on days 7 and 28. The 4-site regimen can therefore be started in a rural clinic with referral a week later. It is economical anywhere if two or more people are treated on the same day.

### Advantages over the 8-site method

The 4-site regimen can be used economically with current vaccines formulated in 0.5 and 1.0 ml ampoule sizes. Our results show that there is no need to divide the initial dose between 8 sites, because it was equally immunogenic in 4 sites. We injected over the deltoid and thigh areas, whereas Ambrozaitis et al. [16] used deltoid and suprascapular sites. The choice might be important in cultures where there is reluctance to expose the thighs.

### Suppression of immune response by concomitant RIG treatment

The efficacy of the 8-site regimen has been demonstrated in patients bitten by proved rabid animals, with and without concomitant RIG [6]. Since the 4-site method has the same timing of doses and amount of vaccine, and is equally immunogenic, it can be inferred that RIG treatment would not be significantly immunosuppressive. All authorities recommend the combination of RIG with vaccine for PEP, especially for high risk exposure to rabies. Treatment failures are inevitable in severe cases (bites on the head, neck or hands or multiple bites) if vaccine is given alone. However RIG is not generally available or affordable in developing countries where it is given to <1% of PEP patients for whom it is recommended [28]. The 4-site regimen fulfils WHO requirements for immunogenicity for PEP and so could be introduced without further studies.

### ID doses

WHO recommendations have changed since 1997, when the difference in dilution was recognised [15], to the latest rule that an ID dose of either vaccine is 0.1 ml [5],[26]. Other studies of 8-site PVRV have used 0.1 ml per ID site [29], as recommended by WHO [5], which almost doubles the amount of vaccine used. The results for the 2-site regimen we report here apply to PVRV, the equivalent dose for PCECV would be 0.2 ml per site.

Ambrozaitis et al. [16] have tested this 4-site regimen to compare different doses of vaccine. Using PCECV, which is formulated in 1 ml ampoule, they showed that 0.1 ml per ID site, a lower dose, was as immunogenic as 0.1 ml per ID site of PVRV. This confirms the safety of our 4-site method, in which 0.25 ml of PCECV would be injected at each ID site on day 0, and 0.2 ml per site subsequently. Using the lower dose of 0.1 ml per site would sacrifice the advantages of using a whole ampoule on the first day, but would be more economical in large treatment centres [13].

### Serological testing

The FAVN and the RFFIT tests are identical in principle but differ in the way their results are read. A comparison between these tests, performed within the same laboratory, showed close correlation [20], but there has been no report of inter-laboratory comparisons. Our data were too few for substantial analysis. In this study, at least one unusually high level was seen with one test, but not confirmed by the other. These results were used in the analysis but did not affect the overall findings or conclusion. Similarly high individual results have been reported previously, but not explained [30],[31],[32]. Rabies immunisation is expensive and unusual in the UK. Thorough investigations excluded previous immunisation in the group analysed and so the high titres cannot be dismissed as an anamnestic response.

Antibody GMTs on days 7 and 14 were much higher, both by the RFFIT and FAVN than in some other recent studies [16],[30]. Over 30 years, no difference has been reported in serological responses to tissue culture rabies vaccines between people in America, Europe and Asia. The higher levels found here remain unexplained. In a 2-site ID vaccine trial in Thailand, antibody levels varied 2.2 fold between different hospitals [30].

### Conclusion

Economical rabies PEP regimens using 2, 4 or 8 initial ID sites are as immunogenic as the standard IM regimen, but they use 60% less vaccine. The 4-site regimen has several practical advantages over both currently used regimens, and is the most economical since only 3 or 4 clinic visits are needed (on days 0, 7 and 28 with optional day 90). Our finding that ID regimens were at least as immunogenic as the “gold standard” 5 dose IM regimen should increase confidence in multiple-site ID techniques. The 4-site regimen is suitable for use anywhere in the world where there are financial constraints, and especially where 2 or more patients are likely to be treated on the same day.

## Supporting Information

Checklist S1 (0.06 MB DOC)

Protocol S1 (0.08 MB DOC)
