# Supplementary material for: A Simplified 4-Site Economical Intradermal Post-Exposure Rabies Vaccine Regimen: A Randomised Controlled Comparison with Standard Methods
Source: PLoS Negl Trop Dis. 2008 Apr 23;2(4):e224. doi: 10.1371/journal.pntd.0000224 (PMC2292256; doi:10.1371/journal.pntd.0000224)
Supplement: Protocol S1 — (0.08 MB DOC) [file pntd.0000224.s002.doc]

THE DEVELOPMENT OF AN IMPROVED ECONOMICAL RABIES POST-EXPOSURE VACCINE REGIMEN

A RANDOMIZED COMPARATIVE STUDY OF THE IMMUNOGENICITY OF MODIFIED MULTISITE INTRADERMAL VACCINE REGIMENS.

##### A Riddell

##### M J Warrell

D A Warrell

H Bourhy

F-X Meslin

##### R Moxon

## The Oxford Vaccine Group Centre for Clinical Vaccinology & Tropical Medicine Churchill Hospital Oxford

Unité de la Rage, Instuit Pasteur, Paris, France

Division of Emerging and Other Communicable DiseaseSurveillance and Control WHO Geneva, Switzerland

*Randomised Comparative study of the Immunogenicity of modified intradermal Post-exposure Rabies vaccine regimens*

*OxREC no.C01.078 (February19^th^ 2004 version 8)*

CONTENTS

Summary

Background and justification

Justification for site of study & volunteers

Objectives

Research design

Procedure

### Recruitment and randomisation of volunteer subjects

### Exclusion Criteria

Informed Consent

New ID regimens

Reference regimens

### Compliance

Serology

Reactogenicity

Statistical analysis

Records and monitoring

References

Table

## Summary

In Asian and African countries, there is a desperate need for effective, safe and economical rabies vaccine for post-exposure prophylaxis. Improved potent and safe tissue culture and purified chick embryo vaccines are now available. They remain too expensive for general use in Asia when given in the conventional regimens demanding a total of 5 ampoules given by intramuscular injection. Multisite intradermal immunization with tissue culture vaccines has proven effective in Thailand, the Philippines and some other Asian countries and has substantially reduced the cost of rabies post-exposure prophylaxis. In most tropical developing countries, rabies hyperimmune globulin is virtually unobtainable and so vaccine must induce virus-neutralizing antibody as rapidly as possible. It is proposed to develop a single economical multisite intradermal regimen for post-exposure prophylaxis, which would induce an accelerated immune response and be suitable for use with all available tissue culture rabies vaccines.

Two new intradermal regimens employing purified vero cell vaccine will be compared with two reference regimens - the "Essen" intramuscular regimen and the "Thai Red Cross" 2-site intradermal regimen in volunteer subjects. The immunogenicity of the regimens will be assessed by virus neutralizing antibody as measured by the rapid fluorescent focus inhibition test (RIFFT). It is proposed that the study should be carried out in volunteers in the UK. Successful development of a single economical regimen for post-exposure prophylaxis, suitable for use with all available vaccines under all circumstances, would be of great practical use in tropical developing countries especially in Asia and Africa. If it were shown to be as immunogenic as the intramuscular regimen, the new method would be applicable worldwide.

**Background and justification**

Worldwide about 10 million people each year receive rabies vaccine after exposure, usually by a dog bite, to the risk of contracting rabies. Many of these people are given vaccines prepared in animal brain tissues (from sheep, goat or mice), the efficacy and safety of which is questionable. In spite of treatment 30,000 people are reported to die of rabies each year in India alone. Many cases remain unreported and it is estimated that the global number of human rabies cases may be 60,000 each year, the majority occurring in Asia. Many of the deaths result from lack of vaccine of adequate quality, a consequence of the high current price of US$50-80 (£UK 90) for a five dose conventional course of intramuscular (IM) post-exposure vaccine treatment (PET) in Asia. In most families living in rabies endemic areas of Africa, Asia or Latin America, a potentially rabid dog bite is a major financial crisis as they often cannot afford PET, which costs about four times their per capita annual health expenditure.

Alternative routes and dosages of modern rabies vaccines were therefore explored. The intradermal (ID) route for the application of small doses of vaccine (0.1 ml) was first tested for safety and efficacy in Thailand in the mid-1980s. These use one-tenth to one-fifth of a single IM dose of modern cell culture rabies vaccine. The 8-site regimen was tested with human diploid cell vaccine, ^1-4^ and then the 2-site regimen with purified vero cell vaccine (PVRV).^5^  These studies demonstrated the immunogenicity and efficacy of multisite ID vaccine doses and also confirmed that PET by this route would lead to savings of at least 60% compared to the cost of conventional PET using five full doses (0.5 or 1 ml) of vaccine by the IM route. Consultants at the Veterinary Public Health Division of WHO assessed the safety and efficacy of the ID technique. Both the 8-site (804011) ^3^ and the 2-site (222011)^5^ ID regimens were selected and guidelines for their implementation were published.^6^ These two economical regimens have been used in over 100,000 patients in Thailand, the Philippines and India.

Widespread implementation of the methods has been hampered by the manufacture of doses of vaccines in different volumes, either 0.5 or 1.0 ml per dose. ID regimens are therefore not readily applicable with all products. Confusion has ensued, the instructions are complicated and the risk of errors cannot be ignored. This study aims to simplify treatment. The 8-site regimen has several advantages, including safety, ^7,8^ but the vaccine which dominates the international market, purified vero cell vaccine (PVRV), has not been tested with this regimen. It is possible that dividing the vaccine between 4 sites ID will be as effective and more convenient than at 8 sites.

**Justification for site of study and volunteer subjects**

Successful development of a single economical regimen for post-exposure prophylaxis, which would be suitable for use with all available vaccines under all circumstances, would be of great practical use in tropical developing countries especially in Asia and Africa. If the new method were shown to be as immunogenic as the intramuscular regimen, it would be applicable worldwide. All previous studies of ID PET regimens have been conducted in dog rabies endemic countries of Asia, resulting in criticisms that it is a ‘second class’ treatment, and a reluctance to trust its efficacy. The confidence of the method might be increased in peoples of the developing countries by testing and promoting ID techniques in Europe. Students, health workers, the general public or military volunteers will receive a licensed vaccine and blood samples will be taken over a year. They will then have had excellent pre-exposure prophylaxis. Should they be exposed to rabies in the future, vaccine will be needed but not rabies immune globulin treatment. An enormous accumulated clinical experience with the European vaccines suggests that the risks to the vaccinees are absolutely minimal.

**Objectives:**

To find a single economical post-exposure rabies vaccine regimen suitable for use with all vaccines currently recommended by the WHO, by testing the initial immunogenicity of two new variations of current ID PET regimens. Any new method must induce a rapid initial immune response, in comparison with control regimens.

**Research design**

This project will study the immunogenicity of two new ID regimens in volunteers who have previously never been immunized against rabies. The new ID regimens will require a similar amount of vaccine as the control ID regimens (a total of less than two full intramuscular doses) and only three or four visits to the vaccination clinic (days 0, 7, 28, 90). The study will be carried out by staff of the the Oxford Vaccine Group Centre for Clinical Vaccinology & Tropical Medicine at the Churchill Hospital, Oxford.

Evaluation of the immunogenicity of two new ID regimens in comparison with known safe and efficacious reference regimens and vaccines

In a randomised study the regimens under test should be shown to induce an adequate level of virus-neutralizing antibody (VNA) measured by RFFIT performed according to the WHO protocol comparable in time and titre to that shown for control reference regimens of proven efficacy.

The vaccine will be Purified Vero Cell Rabies Vaccine (PVRV) 0.5 ml/ vial of known potency manufactured by Aventis (the Medicines and Healthcare Products Regulatory Agency has granted exemption from a licence).

## Procedure

### Recruitment and randomisation of volunteer subjects

Recruitment will be by posters, information leaflets and radio or newspapers. Subjects who respond to the above will be sent the information letter. Those who respond either by letter or by phone will be contacted by study staff and an appointment made with the study team. 220 healthy volunteers in the UK between the ages of 18 and 50 years will be recruited. They will be randomised into one of 4 treatment groups of 55 people each. Randomisation is by a computer generated block scheme for treatment allocation using Stata program, designed so that reducing the number of groups and sample size would not affect the randomisation. The data will be concealed in sequential envelopes, and opened after the consent form has been signed. The follow up will last for one year. The volunteers’ General Practitioners will be informed.

### Exclusion Criteria

Any previous rabies immunisation, treatment with human immunoglobulins or blood transfusion within the past 3 months, the use of immunosuppressive drugs, pregnancy and uncertainty about returning for appointments during the year. Chloroquine cannot be taken for two weeks prior to vaccination at day 0 until 2 weeks after vaccination at day 90.

### Informed Consent

Adult volunteers will be sent an information sheet about the study and asked if they would like to take part. At the initial visit, prior to obtaining consent, a doctor or nurse on the research team will give a full explanation of the study protocol to the volunteer. Having given written consent to participation, the volunteer will then be randomised (see method above) to receive one of the following regimens.

New ID regimens (see Table)

A **PVRV** **4-site** regimen "40201" which consists of one dose of 0.5 ml divided between four sites (deltoid and thigh areas) on day 0. The entire dose is drawn into one syringe and approximately one fourth the dose given at each site. The entire contents of the vial (0.5ml) must be given. The regimen is completed by 0.1ml at two sites (deltoid) on day 7 and one injection of 0.1 ml on days 28 and 90. (This uses the principle of the 8-site method^3^ adapted to a vaccine where the ampoule contains 0.5 ml, using the same dose per site as the 2-site method.^6^).

B **PVRV 8-site** regimen "80401" which consists of one dose of 0.5 ml divided between eight sites (deltoid, suprascapular, lower abdominal wall and thigh areas) on the first day. Note that the entire dose is drawn up into one syringe and approximately one eighth of the total volume is given intradermally at each site. The entire contents of the vial (0.5ml) must be given. The regimen is completed by one ID injection of 0.05 ml at each of four sites (deltoid and thigh areas) on day 7, and one injection at one site (deltoid) of 0.05 ml on days 28 and 90. (This is the current 8-site method used with an ampoule size of 0.5 ml.)

# Reference regimens

For comparison, two widely used vaccine regimens will be included:

D **PVRV** **2-site** ID "Thai Red Cross" regimen "22201": Two ID doses of 0.1 ml each are given over the deltoid on days 0, 3 and 7, with a single site ID injection on days 28 and 90.

E Standard **IM** regimen ("Essen regimen") : A dose of 0.5ml PVRV given IM into the deltoid on days 0, 3, 7, 14 and 28.

### Compliance

Blood samples must be taken on the correct day up to day 14. The permitted deviations are: on day 28, ± 4 days, on day 90, -7 to +10 days, and at one year 2 weeks before to 4 weeks after the date. However it is hoped that the precise date will be adhered to.

### Serology

A 10 ml sample of venous blood will be taken on **days 0, 7, 14, and 90 and at 1 year**, in total five samples per person. Blood will be centrifuged within 24 hours, the serum coded and 2 aliquots stored separately at –70° C. VNA will be measured by the Rapid Fluorescent Focus Inhibition Test (RFFIT), or the fluorescent antibody neutralisation (FAVN) test, at the Pasteur Institute in Paris.

Reactogenicity

At each visit the subjects will be given a reactogenicity diary to complete. They will be asked to document daily temperatures, local and general reactions and any other adverse events, post vaccination. Subjects will be requested to report serious adverse events directly to the study team.

Statistical Analysis

The aim of the study is to show that the test regimens are at least as protective as the standard regimens, i.e. to demonstrate one-sided equivalence. The outcome is the proportion of subjects reaching an internationally accepted minimum VNA level of > 0.5 IU/ml by day 14. The failure rate for the current regimen in meeting this threshold is less than one in 1000. On the basis of this rate, the expected number of failures in the control group is likely to be zero. The sample size calculation has been undertaken assuming that the new regimen is just as effective (i.e. rate of less than 1 in 1000). The trial is also making comparisons between more than 2 groups, so it is necessary to make adjustments to significance levels for the number of comparisons made. Standard methods of calculating sample size estimates cannot be used in this situation as the failure rates are too low, and the study must demonstrate equivalence. The analysis will concentrate on estimating the confidence interval for the difference, which will be done by exact methods due to the expected low frequency of events. The problem has been approached by randomly generating a representative set of trials.

A total of 6 comparisons will be made among the four groups (i.e. A vs B, A vs C, A vs D, B vs C, B vs D, C vs D). If no events are observed in any of the pairwise comparisons, we can be 95% certain that the new regimens have efficacy within 9% of the control regimens when 55 subjects per group are recruited, with power of 80%. The margin of non-inferiority suggested by FDA is not more than 10%.

Records and monitoring

All hard copy data will be kept by the Oxford Vaccine Group (OVG) in secure study records, and all electronic data will be placed on a server set up for the purpose of keeping confidentiality. Access will only occur through a password that is restricted to those involved in the study. No individuals will be identifiable from published results without their explicit consent. The data will be monitored internally by OVG.

## References

1. Warrell MJ, Warrell DA, Suntharasamai P, Viravan C, Sinhaseni A, Udomsakdi D, Phanfung R, Xueref C, Vincent-Falquet J-C, Nicholson KG, Bunnag D, and Harinasuta T. (1983). An economical regimen of human diploid cell strain anti-rabies vaccine for post-exposure prophylaxis. *Lancet* ii 301-4.
2. Warrell MJ, Suntharasamai P, Nicholson KG, Warrell DA, Chantavanich P, Viravan C, Sinhaseni A, Phanfung R, Xueref C, Vincent-Falquet J-C (1984). Multi-site intradermal and multi-site subcutaneous rabies vaccination: improved economical regimens. *Lancet* i 874-876.
3. Warrell MJ, Nicholson KG, Warrell DA, Suntharasamai P, Chantavanich P, Viravan C, Sinhaseni A, Chiewbamroongkiat M, Pouradier-Duteil X, Xueref C, Phanfung R, Udomsakdi D. (1985). Economical multiple-site intradermal immunisation with human diploid-cell-strain vaccine is effective for post-exposure rabies prophylaxis. *Lancet* i 1059-62.
4. Suntharasamai P, Warrell MJ, Warrell DA, Chantavanich P, Looareesuwan S, Supapochana A, Phanuphak P, Jittapalapongsa S, Yager P, Baer G. (1987). Early antibody responses to rabies post-exposure vaccine regimens*. Amer J Trop Med Hyg* 36 (1) 160-5.
5. Chutivongse S, Wilde H, Supich C. Baer GM, Fishbein DB (1990). Post exposure prophylaxis for rabies with antiserum and intradermal vaccination. *Lancet* 335 896-8.

6 WHO Recommendations on rabies post-exposure treatment and the correct technique of intradermal immunization against rabies. WHO 1997 WHO/EMC/ZOO.96.6.

7 Madhusudana SN, Anand NP, Shamsundar R. Evaluation of two intradermal vaccination regimens using purified chick embryo cell vaccine for post-exposure prophylaxis of rabies. *Natl Med J India* 2001;**14**(3)**:**145-7.

8 Warrell MJ. The challenge to provide affordable rabies post-exposure

treatment. Vaccine. *Vaccine* 2003;**21:**706-9.

**Table : REGIMENS TO BE TESTED**

#### Vaccine Regimen Day 0 3 7 14 28 90 1 year

# New ID regimens

**A** PVRV "40201" 0.5ml divided 0 2 x 0.1 ml 0 1 x 0.1 ml 1 x 0.1 ml

between 4 sites

**B**. PVRV "80401" 0.5ml divided 0 4 x 0.05 0 1 x 0.05 1 x 0.05

between 8 sites

# Reference regimens

**D.** PVRV "22201" 2 x 0.1 2 x 0.1 2x 0.1 0 1 x 0.1 1 x 0.1

**E.** PVRV (Essen IM) 1 x 0.5 1 x 0.5 1 x 0.5 1 x 0.5 1 x 0.5 0

VNA blood test  **+ + + + +**
